# Super-flat supercontinuum generation from a Tm-doped fiber amplifier

**DOI:** 10.1038/srep23759

**Published:** 2016-03-29

**Authors:** Mengmeng Tao, Ting Yu, Zhenbao Wang, Hongwei Chen, Yanlong Shen, Guobin Feng, Xisheng Ye

**Affiliations:** 1 State Key Laboratory of Laser Interaction with Matter, Northwest Institute of Nuclear Technology, Xi’an 710024, P. R. China; 2 Research Center of Space Laser Information Technology, Shanghai Institute of Optics and Fine Mechanics, Chinese Academy of Sciences, Shanghai 201800, P. R. China

## Abstract

Super-flat supercontinua are generated from a double clad Tm-doped fiber amplifier. Two different laser configurations are investigated and compared. In the direct-output configuration, the long-wavelength edge of the supercontinuum spectra is extended to beyond 2.65 μm with a 10 dB bandwidth of 740 nm. In the passive pigtail configuration, the generated supercontinuum features excellent flatness with an intensity difference smaller than 1 dB in the wide central spectral range from 1.98 μm to 2.41 μm.

Supercontinuum has been studied for decades as its broadband spectral characteristics promise enormous potentials in many significant research fields and practical applications^1^,^2^,^3^. Up to now, many different supercontinuum sources have been developed with different pump sources, such as Yb-doped fiber lasers^4^,^5^ and Er-doped fiber laser^6^,^7^,^8^,^9^, in various fibers including conventional single mode fibers^3^,^9^, GeO_2_ fibers^10^,^11^,^12^, highly nonlinear fibers^13^,^14^, photonic crystal fibers^5^,^15^,^16^ and ZBLAN fibers^17^,^18^,^19^,^20^,^21^.

Besides, active fibers, especially Tm-doped fibers, have also been found to be an effective medium for supercontinuum generation^8^,^9^,^10^,^22^,^23^,^24^. And, along with the fast development of Tm-doped fiber lasers, 2 μm laser sources have demonstrated its unmatchable advantages as an ideal pump in the generation of mid-infrared supercontinuum^17^,^18^,^19^,^20^,^21^. As Tm-doped fiber could be used not only as the gain medium for 2 μm laser source but also as the nonlinear medium for supercontinuum generation, a Tm-doped fiber based supercontinuum system would be preferable. In Tm-doped fiber based supercontinuum systems, the supercontinuum generation process exhibits more complicated physical mechanisms from passive fibers as the ^3^F_4_-^3^H_6_ and ^3^H_4_-^3^H_5_ transitions in Tm ions also play an important role in the spectral broadening^8^,^22^,^23^,^24^,^25^.

As early as in 2007, S. Kivisto and his colleagues reported the supercontinuum generation in Tm/Ho codoped fiber amplifiers spanning from 1.95 μm to 2.25 μm^26^. In 2013, J. Liu demonstrated a high power supercontinuum source from a three-stage Tm-doped fiber amplifier, and the spectral range is extended to beyond 2.4 μm^27^. Later, in 2014, V. V. Dvoyrin extended the long-wavelength side to 2.5 μm with only one-stage amplifier^28^. However, in these reports, the flatness of the supercontinua generated is not satisfying with intensity differences of about 10 dB.

In our previous works, pulsed 2 μm laser sources were built^29^,^30^,^31^. In this report, exploiting the self-developed 2 μm laser source as the seed, together with a 793 nm LD as the pump, a Tm-doped fiber amplifier for supercontinuum generation is developed. And, two different supercontinuum generation configurations, namely the direct-output configuration and the passive pigtail configuration, are investigated. In the direct-output configuration, the laser output is directly measured at the output end of the double clad Tm-doped fiber. Differently, in the pigtail configuration, a section of passive fiber is spliced at the output end of the double clad Tm-doped fiber. And, the laser output is recorded at the angle cleaved output end of the passive fiber. Supercontinuum is observed in both schemes, but with different characteristics. In the direct-output configuration, the 3 dB bandwidth of the supercontinuum generated reaches about 600 nm, while, in the pigtail configuration, a super-flat supercontinuum is attained with an intensity difference of only 0.87 dB in the wide central spectral range from 1.98 μm to 2.41 μm.

## Results

In the laser system, the pulsed 2 μm laser seed exploited has a maximal output power of 1.1 W with the repetition rate tunable from 20 kHz to 100 kHz. At 20 kHz, repetition rate, the seed operates in the gain-switched mode-locking mode^29^,^30^,^31^ where the 100 ns gain-switched envelope contains tens of sub-pulses with durations of about 8 ns. At higher repetition rates, the seed works in the gain-switching mode with a pulse duration of about 50 ns. The amplification fiber is a section of 7 m long double clad Tm-doped fiber (Nufern, 10P/130) with an absorption coefficient of about 3 dB/m at 793 nm. The hexagonal fiber core has an NA of 0.15 and a core diameter of 10 μm. The cladding diameter of the fiber is 130 μm.

**Figure 1:**
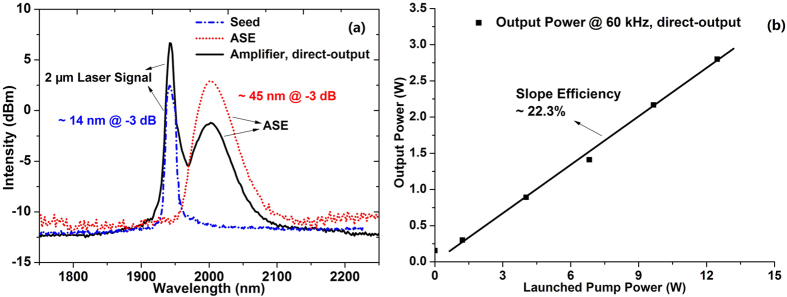
Output characteristics of the double clad Tm-doped fiber amplifier at 60 kHz repetition rate: (**a**) output spectrum of the amplifier; (**b**) output power versus the launched pump power.

Firstly, output characteristics of the double clad Tm-doped fiber amplifier in the direct-output configuration at 60 kHz repetition rate are investigated. And, the results are given in Fig. 1 with Fig. 1(a) presenting the laser spectrum and Fig. 1(b) depicting the output power.

The short dashed in Fig. 1(a) shows the measured spectrum of the pulsed 2 μm laser seed after propagation through the 7 m long double clad Tm-doped fiber. And, the dotted and solid line represent the amplified spontaneous emission (ASE) spectrum of the Tm-doped fiber and the output spectrum of the amplifier at 4 W 793 nm pump, respectively. As can be found, the seed spectrum centers around 1.95 μm with a 3 dB linewidth of about 14 nm, the ASE centers at 2 μm with a 3 dB bandwidth of about 45 nm, while the amplifier spectrum is the combination of the two. It should be noted that both the seed spectrum and the ASE spectrum come from the ^3^F_4_-^3^H_6_ transition in Tm ions^25^. The slope efficiency of the amplifier, shown in Fig. 1(b), is about 22.3% with a maximal output of 2.8W at 12.5 W pump, which is comparable with that in ref. 32.

In the pigtail configuration, the amplifier shows the same output characteristics at 60 kHz repetition rate.

**Figure 2:**
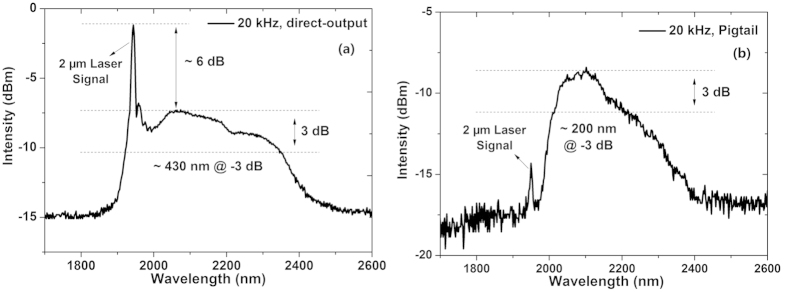
Supercontinuum generation from the Tm-doped fiber amplifier at 20 kHz repetition rate with no 793 nm pump power: (**a**) direct-output configuration with no passive fiber at the output end of the Tm-doped fiber; (**b**) pigtail configuration with 1 m long passive fiber at the output end of the Tm-doped fiber.

Tuning the repetition rate of the 2 μm laser seed to 20 kHz, supercontinuum is generated in the Tm-doped fiber amplifier in both laser configurations even with no 793 nm pump, as illustrated in Fig. 2. Figure 2(a) presents the supercontinuum generated from the Tm-doped fiber amplifier with the direct-output configuration where there is no passive fiber at the output end of the double clad Tm-doped fiber, while Fig. 2(b) gives the supercontinuum generated from the Tm-doped fiber amplifier with the passive pigtail configuration where a section of 1 m long SMF28 passive fiber is spliced at the output end of the double clad Tm-doped fiber.

**Figure 3:**
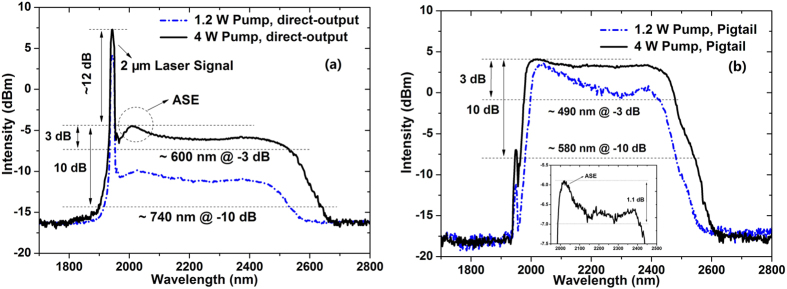
Supercontinuum generation from the Tm-doped fiber amplifier at 20 kHz repetition rate with different 793 nm pump powers: (**a**) direct-output configuration; (**b**) pigtail configuration.

More supercontinua are recorded in Fig. 3 at different 793 nm pump powers. Figure 3(a) provides the laser spectra measured in the direct-output configuration at different 793 nm pump powers. And, laser spectra measured from the passive pigtail configuration are depicted in Fig. 3(b).

**Figure 4:**
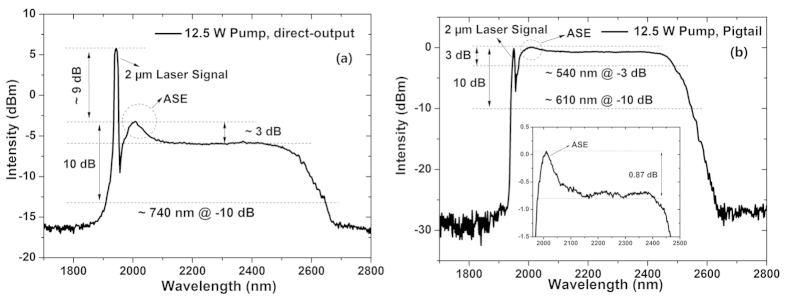
Maximal supercontinuum generated from the Tm-doped fiber amplifier at 20 kHz repetition rate with 12.5 W 793 nm pump: (**a**) direct-output configuration; (**b**) pigtail configuration.

**Figure 5:**
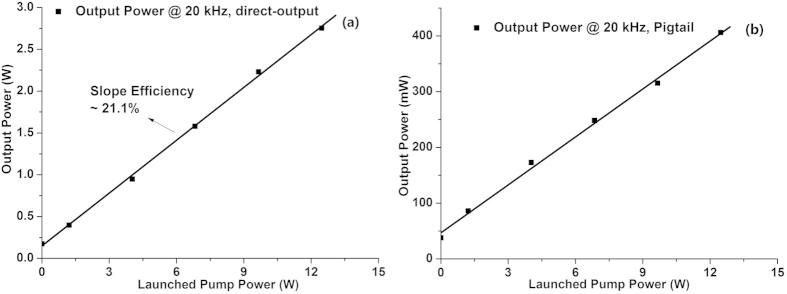
Output power of the Tm-doped fiber amplifier at 20 kHz repetition rate: (**a**) direct-output configuration; (**b**) pigtail configuration.

The supercontinua generated at maximal pump power are shown in Fig. 4. And, output powers of the Tm-doped fiber amplifier is presented in Fig. 5.

## Discussion

The output spectrum in Fig.1 (a) demonstrates that the 2 μm laser signal is not effectively amplified as ASE is generated during the amplification process.

As can be found in Fig. 2(a), the supercontinuum generated with no passive pigtail characterizes a wideband long-wavelength supercontinuum region with a 3 dB bandwidth of about 430 nm covering a wide wavelength range from 1.92 μm to 2.35 μm and a strong unabsorbed 2 μm laser signal which is about 6 dB higher than the long-wavelength supercontinuum. This phenomenon is also observed in refs 17, 18, 19, 20, 21. Different from that in Fig. 2(a), with the passive fiber, the 2 μm laser signal in Fig. 2(b) becomes much smaller. However, the spectrum becomes narrower with a 3 dB bandwidth of about 200 nm centering around 2.1 μm. This wide spectral peak around 2.1 μm is also observed in Fig. 2(a). We believe that this should be attributed to the ^3^H_4_-^3^H_5_ transition in Tm ions^8^,^22^,^23^,^24^.

In addition, both spectra in Fig. 2 mainly broaden towards the long-wavelength side which is quite different from those in in 1.6 μm pumped supercontinuum generation where the supercontinua generated broaden on both spectral sides^6^,^7^,^24^. The main reason for the generation of the red-shifted supercontinuum is modulation instability which leads to soliton fission and soliton self-frequency shift induced by Raman scattering^9^,^18^,^19^,^24^.

Similar with that in Fig. 2(a), the relatively strong 2 μm laser signal remains at different 793 nm pump powers in Fig. 3(a). Besides, ASE signal is observed around 2 μm. At 4 W pump, the 3 dB bandwidth of the supercontinuum generated broadens to about 600 nm, while the 10 dB bandwidth reaches 740 nm with the long-wavelength extending to beyond 2.6 μm. It can be found in Fig. 3(b) that, at 4 W 793 nm pump, the flat supercontinuum demonstrates a 3 dB bandwidth of 490 nm, more than doubled compared to that with no 793 nm pump in Fig. 2(b). Besides, the 2 μm laser signal gets almost eliminated through the propagation in the passive fiber, contributing to the flatness of the generated supercontinua which shows an intensity difference of only 1.1 dB from 1.99 μm to 2.40 μm as depicted in the inset of Fig. 3(b). And, it is obvious that the intensity difference is mainly caused by the strong ASE signal around 2 μm.

As marked in Fig. 4(a), at 12.5 W 793 nm pump, compared with the spectrum at 4 W 793 nm pump power, the bandwidth of the spectrum in the direct-output configuration is not further broadened , while the ASE signal gets stronger which is about 3 dB higher than the long-wavelength supercontinuum. On the contrary, in the passive pigtail configuration, the supercontinuum grows broader with the increase of the pump power, as shown in Fig. 4(b). The 3 dB and 10 dB bandwidths of the supercontinua in passive pigtail configuration reach 540 nm and 610 nm, respectively. And, the intensity difference of the central region of the supercontinuum between 1.98 μm and 2.41 μm is only 0.87 dB which is mainly caused by the strong ASE signal as depicted in the inset of Fig. 4(b), demonstrating excellent flatness of this laser system.

As plotted in Fig. 5(a), when there is no pigtail fiber, the maximal output power is about 2.7 W with a slope efficiency of about 21.1% which is very close to that at 60 kHz in Fig. 1(b), demonstrating that the output power is mainly the power of the 2 μm laser signal. Differently, in the passive pigtail configuration, the output power of the Tm-doped fiber amplifier is mainly the power of the supercontinuum since the 2 μm laser signal gets almost eliminated through the propagation in the passive fiber, as shown in Figs 3(b) and 4(b). However, the output power of the supercontinuum is much smaller with the maximal output of only 400 mW as shown in Fig. 5(b).

## Conclusion

Super-flat supercontinua are generated from a Tm-doped fiber amplifier although the amplification of the seed is not ideal with the attendance of a strong ASE signal. Comparisons between different pump powers and laser configurations are discussed and analyzed. Compared with the passive pigtail configuration, the direct-output configuration enjoys a broader laser spectrum, while the passive pigtail configuration boasts a much flatter supercontinuum. This laser system would be applicable in broadband absorption spectroscopy applications. However, the power of the supercontinuum generated is still relatively low. And, further improvement in the output power and spectral extension should be considered.

## Methods

**Figure 6:**
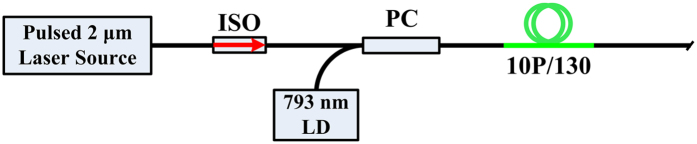
Schematic diagram of the Tm-doped fiber amplifier.

The experimental setup of the Tm-doped fiber amplifier is depicted in Fig. 6. The pulsed 2 μm laser source takes a similar scheme to that described in ref. 25. The 2 μm laser source and the pump source from a 793 nm LD are coupled into a section of 7 m long double clad Tm-doped fiber (Nufern, 10P/130) through a pump combiner (PC).

The laser spectra are recorded with an Andor Shamrock 750 spectrum analyzer. It should be noted that the resolution of the spectrum analyzer is about 2 nm determined by the grating and the step length. And, a thermoelectrically cooled fast HgCdTe detector and a 4 GHz oscilloscope (Tektronix TDS 7404) are exploited to monitor the laser pulses.

## Additional Information

**How to cite this article**: Tao, M. *et al*. Super-flat supercontinuum generation from a Tm-doped fiber amplifier. *Sci. Rep*. **6**, 23759; doi: 10.1038/srep23759 (2016).

## References

[b1] Alfano R. R. & Shapiro S. L. Observation of self-phase modulation and small-scale filaments in crystals and glasses. Phys. Rev. Lett. 24, 592–594 (1970).

[b2] Genty G., Coen S. & Dudley J. M. Fiber supercontinuum sources. J. Opt. Soc. Am. B 24(8), 1771–1785 (2007).

[b3] Kaminski C. F., Watt R. S., Elder A. D., Frank J. H. & Hult J. Supercontinuum radiation for applications in chemical sensing and microscopy. Appl. Phys. B 92, 367–378 (2008).

[b4] Song R. . Near-infrared supercontinuum generation in an all-normal dispersion MOPA configuration abover one hundred watts. Laser Phys. Lett. 10, 015401 (2013).

[b5] Hernandez-Garcia J. C. . Experimental study on a broad and flat supercontinuum spectrum generated through a system of two PCFs. Laser Phys. Lett. 10, 075101 (2013).

[b6] Swiderski J., Theberge F., Michalska M., Mathieu P. & Vincent D. High average power supercontinuum generation in a fluoroindate fiber. Laser Phys. Lett. 11, 015106 (2014).

[b7] Xia C. . Power scalable mid-infrared supercontinuum generation in ZBLAN fluoride fibers with up to 1.3 watts time-averaged power. Opt. Express 15(3), 865–871 (2007). 19532312 10.1364/oe.15.000865

[b8] Geng J., Wang Q. & Jiang S. High-spectral-flatness mid-infrared supercontinuum generated from a Tm-doped fiber amplifier. Appl. Optics 51(7), 834–840 (2012). 10.1364/AO.51.000834 22410883

[b9] Swiderski J. & Michalska M. The generation of a broadband, spectrally flat supercontinuum extended to the mid-infrared with the use of conventional single-mode fibers and thulium-doped single-mode fibers pumped by 1.55 μm pulses. Laser Phys. Lett. 10, 015106 (2013).

[b10] Anashkina E. A., Andrianov A. V., Koptev M. Yu., Muravyev S. V. & Kim A. V. Generating femtosecond optical pulses tunable from 2 to 3 μm with a silica-based all-fiber laser system. Opt. Lett. 39(10), 2963–2966 (2014). 24978248 10.1364/OL.39.002963

[b11] Kamynin V. A. . Supercontinuum generation beyond 2 μm in GeO_2_ fiber: comparison of nano- and femtosecond pumping. Laser Phys. Lett. 12, 065101 (2015).

[b12] Kamynin V. A., Kurkov A. S. & Mashinsky V. M. Supercontinuum generation up to 2.7 μm in the germanate-glass-core and silica-glass-cladding fiber. Laser Phys. Lett. 9(3), 219–222 (2012).

[b13] Zhu H. . Optical stealth transmission based on super-continuum generation in highly nonlinear fiber over WDM network. Opt. Lett. 40(11), 2561–2563 (2015). 26030557 10.1364/OL.40.002561

[b14] Quang N., Matsuura M. & Kishi N. WDM-to-OTDM conversion using supercontinuum generation in a highly nonlinear fiber. IEEE Photonic. Tech. L. 26(18), 1882–1885 (2014).

[b15] Tripathy S. K., Achary J. S. N., Muduli N. & Palai G. Nonlinear rectangular photonic crystal fiber (PCF) for optical communication exclusively super continuum generation. J. Laser Opt. Photonics 2(1), 1000114 (2015).

[b16] Jiang X. . Deep-ultraviolet to mid-infrared supercontinuum generated in solid-core ZBLAN photonic crystal fibre. Nat. Photonics 9, 133–139 (2015).

[b17] Eckerle M. . Actively Q-switched and mode-locked Tm-doped silicate 2 μm fiber laser for supercontinuum generation in fluoride fiber. Opt. Lett. 37(4), 512–514 (2012). 22344090 10.1364/OL.37.000512

[b18] Swiderski J., Michalska M. & Maze G. Mid-IR supercontinuum generation in a ZBLAN fiber pumped by a gain-switched mode-locked Tm-doped fiber laser and amplifier system. Opt. Express 21(7), 7851–7857 (2013). 23571875 10.1364/OE.21.007851

[b19] Yang W., Zhang B. Yin K. Zhou X. & Hou J. High power all fiber mid-IR supercontinuum generation in a ZBLAN fiber pumped by a 2 μm MOPA system. Opt. Express 21(17), 19732–19742 (2013). 24105521 10.1364/OE.21.019732

[b20] Yang W., Zhang B., Hou J., Yin K. & Liu Z. A novel 2 μm pulsed fiber laser based on a supercontinuum source and its application to mid-infrared supercontinuum generation. Chin. Phys. B 23(5), 054208 (2014).

[b21] Heidt A. M. . Mid-infrared ZBLAN fiber supercontinuum source using picosecond diode-pumping at 2 um. Opt. Express 21(20), 24281–24287 (2013). 24104337 10.1364/OE.21.024281

[b22] Kurkov A. S. . Supercontinuum generation in thulium-doped fibers. Quantum Electron. 42(9), 778–780 (2012).

[b23] Yang W. Q. . Mid-IR supercontinuum generation in Tm/Ho codoped fiber amplifier. Laser Phys. Lett. 10, 055107 (2013).

[b24] Swiderski J. & Michalska M. Mid-infrared supercontinuum generation in a single-mode thulium-doped fiber amplifier. Laser Phys. Lett. 10, 035105 (2013).

[b25] Jackson S. D. The spectroscopic and energy transfer characteristics of rare earth ions used for silicate glass fibre lasers operating in the shortwave infrared. Laser Photonics Rev. 3(5), 466–482 (2009).

[b26] Kivisto S., Hakulinen T., Guina M. & Okhotnikov O. G. Tunable Raman soliton source using mode-locked Tm-Ho fiber laser. IEEE Photon. Technol. Lett. 19(12), 934–936 (2007).

[b27] Liu J. Xu J. Liu K. Tan F. & Wang P. High average power picosecond pulse and supercontinuum generation from a thulium-doped, all-fiber amplifier. Opt. Lett. 38(20), 4150–4153 (2013). 24321946 10.1364/OL.38.004150

[b28] Dvoyrin V. V. & Sorokina I. T. 6.8 W all-fiber supercontinuum source at 1.9-2.5 μm. Laser Phys. Lett. 11, 085108 (2014).

[b29] Tao M. . Experimental investigation of gain-switched Tm-Ho Co-doped single clad fiber lasers. Laser Phys. 23, 105101 (2013).

[b30] Tao M. . Gain-switching and gain-switched mode-locking operation of a Tm/Ho co-doped fiber laser. Laser Phys. 23, 095109 (2013).

[b31] Tao M. . Tm-Ho codoped fiber based all fiber amplification of a gain-switched 2 μm fiber laser. Optik 125, 6198–6200 (2014).

[b32] Yang W. Q. . Gain-switched and mode-locked Tm/Ho-codoped 2 μm fiber laser for mid-IR supercontinuum generation in a Tm-doped fiber amplifier. Laser Phys. Lett. 10, 045106 (2013).

